# Face identity matching is influenced by emotions conveyed by face and body

**DOI:** 10.3389/fnhum.2014.00053

**Published:** 2014-02-12

**Authors:** Jan Van den Stock, Beatrice de Gelder

**Affiliations:** ^1^Department of Neuroscience, Division of Old Age Psychiatry, Brain and Emotion Laboratory Leuven (BELL)KU Leuven, Leuven, Belgium; ^2^Old Age Psychiatry, University Hospitals LeuvenLeuven, Belgium; ^3^Cognitive and Affective Neuroscience Laboratory, Tilburg UniversityTilburg, Netherlands; ^4^Department of Cognitive Neuroscience, Faculty of Psychology and Neuroscience, Brain and Emotion Laboratory Maastricht, Maastricht UniversityMaastricht, Netherlands

**Keywords:** face, body, emotion, identity, context

## Abstract

Faces provide information about multiple characteristics like personal identity and emotion. Classical models of face perception postulate separate sub-systems for identity and expression recognition but recent studies have documented emotional contextual influences on recognition of faces. The present study reports three experiments where participants were presented realistic face-body compounds in a 2 category (face and body) × 2 emotion (neutral and fearful) factorial design. The task always consisted of two-alternative forced choice facial identity matching. The results show that during simultaneous face identity matching, the task irrelevant bodily expressions influence processing of facial identity, under conditions of unlimited viewing (Experiment 1) as well as during brief (750 ms) presentation (Experiment 2). In addition, delayed (5000 ms) face identity matching of rapidly (150 ms) presented face-body compounds, was also influenced by the body expression (Experiment 3). The results indicate that face identity perception mechanisms interact with processing of bodily and facial expressions.

## Introduction

Faces provide powerful interpersonal communicative signals and influential theories of face perception have proposed dedicated behavioral and neural mechanisms underlying perception of faces. Two hallmarks of classical theories of face perception are that processing of faces is dominant over other object classes and that different kinds of facial information like identity, expression and direction of gaze are processed in separate, relatively independent subsystems (e.g., Bruce and Young, 1986; Haxby et al., 2000; Calder and Young, 2005). Yet, there is growing evidence challenging these basic principles. For instance, it has been reported that contextual cues that in daily life frequently co-occur with faces influence how we perceive and process faces (de Gelder et al., 2006; de Gelder and Van den Stock, 2011b; Wieser and Brosch, 2012). For example, studies have shown that perception of facial expressions is influenced by vocal expressions (de Gelder and Vroomen, 2000), bodily expressions (Meeren et al., 2005; Van den Stock et al., 2007; Aviezer et al., 2008) and background scenes (Righart and de Gelder, 2006, 2008a; Van den Stock and de Gelder, 2012; Van den Stock et al., 2013b). There is also evidence that facial expressions influence recognition of body expressions (Willis et al., 2011). From a theoretical perspective, these cross-categorical emotional context influences may be explained by activation of an emotion system that is not category specific and therefore common for faces and bodies, thereby modulating face expression categorization.

Secondly, a few studies have challenged the notion of segregated processing streams for identity and expression perception. On the one hand, there is evidence from studies exploiting perceptual mechanisms like interference (Schweinberger and Soukup, 1998; Schweinberger et al., 1999) and adaptation (Leopold et al., 2001; Webster et al., 2004), indicating that recognition of facial expressions interacts with task-irrelevant processing of facial identity, while recognition of identity is relatively independent of facial expression (Fox and Barton, 2007; Fox et al., 2008). On the other hand, using a sequential match-to-sample paradigm, Chen et al. (2011) reported lower accuracies for matching facial identities with emotional expressions, compared to neutral faces, consistent with other studies using different paradigms (D’argembeau et al., 2003; Kaufmann and Schweinberger, 2004; Gallegos and Tranel, 2005; D’argembeau and Van Der Linden, 2007; Savaskan et al., 2007; Levy and Bentin, 2008). In addition, there is clinical evidence from subjects with prosopagnosia that identity perception is influenced by the emotion conveyed by the face (de Gelder et al., 2003; Van den Stock et al., 2008; Huis in ’t Veld et al., 2012).

These studies investigated either contextual influences on face emotion perception or interactions between face identity and face emotion processing. However, little is known about whether contextual emotion cues, such as body postures also influence perception of the facial identity, which is presumably, at least partly processed by different mechanisms than the ones that are the emotional components in the face perception network (Haxby and Gobbini, 2011). In this study, we combine findings of contextual modulation of facial expression perception on the one hand, and face identity and emotion interactions on the other hand. We investigated whether emotional information conveyed by both facial and bodily expressions influences perception of facial identity. For this purpose we created compound images of whole persons consisting of either neutral or emotional faces and bodies that had matched or mismatched expressions while participants were always required to assess the face identity. This design allows contrasting predictions of different theories on facial identity recognition. On the one hand, theories dedicating a cardinal role to processing of the shape of the face (e.g., Kanwisher et al., 1997), would predict minimal influences of both the facial as well as the bodily expression. On the other hand, a significant influence of the emotion of the facial and bodily expression on face identity recognition is more compatible with theories proposing distributed but parallel and interactive processing of multi-faceted faces (e.g., de Gelder et al., 2003; Campanella and Belin, 2007).

## Experiment 1: self-paced simultaneous matching of face identity

### Method

#### Participants

Twenty participants volunteered for the experiment (10 male, mean (SD) age = 23.9 (7.7)) in exchange for course credits. None of the participants had a neurologic or psychiatric history and all had normal or corrected to normal vision. Informed consent was obtained according to the declaration of Helsinki.

#### Stimulus materials

Pictures of facial expressions were taken from the Karolinska Directed Emotional Faces (KDEF) (Lundqvist et al., 1998) and from our own database. In a pilot study, the faces were randomly presented one by one on a screen and participants (*N* = 20) were instructed to categorize the emotion expressed in the face in a seven alternative forced choice paradigm (anger, disgust, fear, happiness, neutral, surprise or sadness). None of these participants took part in any of the other experiments. On the basis of this pilot study, we selected 80 fearful (40 female) and 80 neutral (40 female) facial expressions, all recognized accurately by at least 75% of the participants.

Stimuli of whole body expressions were taken from our own validated database (de Gelder and Van den Stock, 2011a). The selected stimuli displayed fearful body postures and an instrumental action (pouring water in a glass). We used action images instead of neutral body postures, because like the fearful expressions, instrumental actions elicit movement and action representation and we wanted to control for these variables. Forty fearful (20 female) and 40 instrumental (20 female) body expressions were selected.

We created realistic face-body compounds by carefully resizing and combining facial and bodily expressions. A total of 80 compound stimuli were created following a 2 face (fearful and neutral) × 2 body (fearful and neutral) factorial procedure, resulting in 20 stimuli (10 male) per condition. Face and body were always of the same gender, but only half of face-body pairs expressed the same emotion, with the other half displaying an emotion mismatch (e.g., a fearful face with a neutral body).

#### Procedure

**Figure 1:**
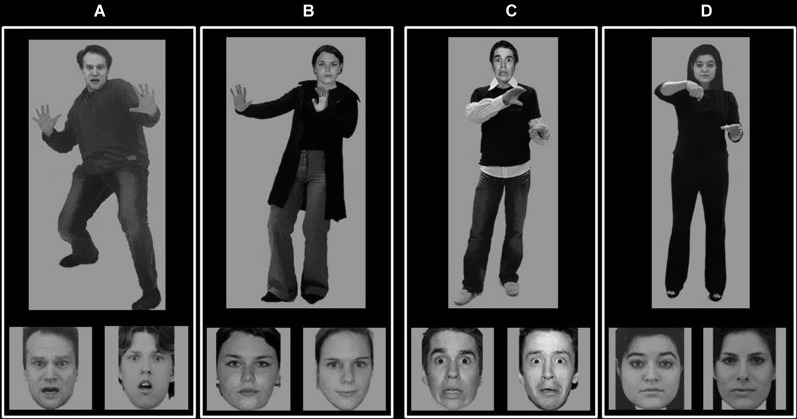
**Stimulus examples**. Examples of experimental stimuli showing on top a fearful face on a fearful body **(A)**; a neutral face on a fearful body **(B)**; a fearful face on a neutral body **(C)** and a neutral face on a neutral body **(D)**. On the bottom two face identities are presented. Both show the same expression as the one on top, but only one is of the same actor as the face on top (in the figure the bottom left alternative is always of the same identity as the one on top).

A trial consisted of a compound face-body stimulus presented simultaneously with two face images left and right underneath the face-body compound image. One of the faces was the same as the face of the compound stimulus. The other face belonged to a different actor, but was matched regarding emotional expression as well as main visual features, such as hair color and gender (see Figure 1 for stimulus examples). Participants were instructed to indicate which of the two bottom faces matched the one of the compound stimulus. We attempted to minimize the visibility of non-face identifying cues, such as hairstyle in both face alternatives. Therefore, both face alternatives only showed the inner canvas of the head, this in order to reduce simple image-matching processes. Instructions stressed to answer as accurately and as quickly as possible. The stimuli were presented until the participant responded. Interstimulus interval was 2000 ms. The experiment started with two practice trials, during which the subject received feedback. The position of the target face was counterbalanced.

### Results

**Figure 2:**
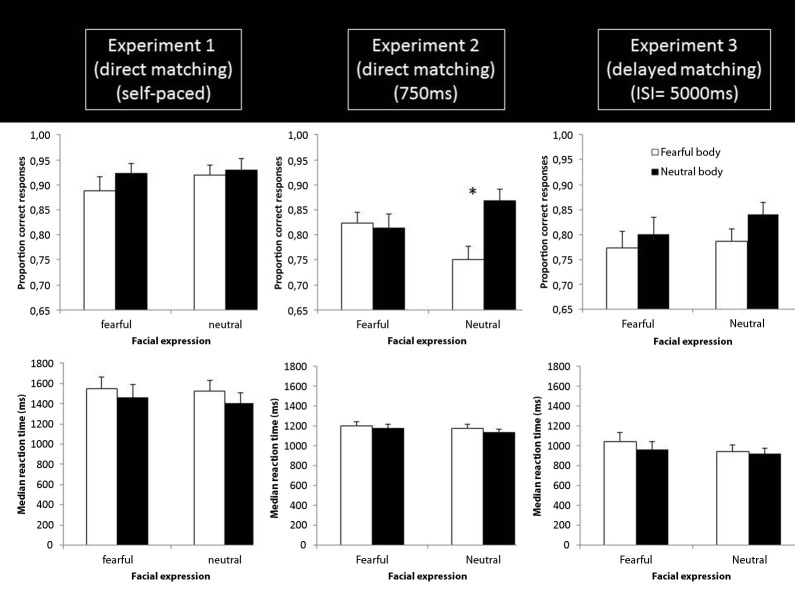
**Results of Experiment 1–3**. Proportion correct identity matching responses (top row) and median reaction times (bottom) as a function of facial and bodily expression in Experiment 1 (left column), Experiment 2 (middle column) and Experiment 3 (right column). ISI: inter-stimulus interval * *p* =0.002.

Mean accuracies and median response times (RTs) were calculated for every condition. The results are shown in the left panel of Figure 2. A 2 facial expression (fearful and neutral) × 2 bodily expression (fearful and neutral) repeated measures ANOVA was carried out on the accuracy and Response time (RT) data. This revealed for the accuracy data a main effect of facial expression (*F*_(1,19)_ = 4.571; *p* = 0.046; $\eta_{p}^{2}=0.194$) and bodily expression (*F*_(1,19)_ = 4.678; *p* = 0.043; $\eta_{p}^{2}=0.198$) , but no significant interaction (*F*_(1,19)_ = 0.812; *p* = 0.379; $\eta_{p}^{2}=0.041$). The main effect of facial expression reflects that neutral faces are matched more accurately than fearful faces, while the main effect of body expression indicates that faces with a neutral body are more accurately matched than faces with a fearful body. The reaction time data only showed a main effect of bodily expression (*F*_(1,19)_ = 12.100; *p* = 0.003; $\eta_{p}^{2}=0.389$), indicating that matching faces with a neutral body was performed faster than matching faces with a fearful body.

There was an equal number of male and female participants in the present experiment, as there is evidence of gender differences in emotion perception (Donges et al., 2012; Kret and de Gelder, 2012). To investigate the influence of gender of the observer on the results, we performed the same repeated-measures ANOVAs with gender of the observer as an additional between subjects variable. This revealed that there were no significant main or interaction effects of gender of the observer (all *p’*s > 0.239). Therefore, we considered gender of the observer as a variable of non-importance in the following experiments.

### Discussion

The results show that matching of facial identity is influenced by the emotion expressed in the face, but also by the task irrelevant body expression as seen in the accuracy and reaction time data. Accuracy and reaction time data show consistent patterns, indicating that the effects cannot be explained by a speed-accuracy trade-off. The lower accuracy for matching identity of fearful faces compared to neutral faces is in line with a recent study using a sequential match-to-sample paradigm (Chen et al., 2011). More interesting for the present purpose: the body expression effect shows that the previously reported influence of body emotion on recognition of facial emotion (de Gelder et al., 2006; de Gelder and Van den Stock, 2011b) extends to facial identity recognition.

Although the instruction stated to respond as accurately and as fast as possible, the viewing time was unlimited. A possible explanation for the body expression effect may be that subjects spent more time looking at the fearful body expressions, compared to the neutral ones. Therefore, a question is whether the body expression effect still obtains with limited viewing time when the duration of stimulus presentation is too short to allow exploration of task irrelevant stimulus attributes. We investigated this issue in Experiment 2.

## Experiment 2: time-constrained simultaneous matching of face identity

### Method

#### Participants

Nineteen participants volunteered for the experiment (2 male, mean (SD) age = 19.2 (1.6)) in exchange for course credits. None of the participants had a neurologic or psychiatric history and all had normal or corrected to normal vision. Informed consent was obtained according to the declaration of Helsinki.

#### Procedure

The procedure was identical to the one in Experiment 1, except that stimulus presentation was limited to 750 ms. A pilot study with different durations indicated that 750 ms was the shortest duration that was still associated with an acceptable accuracy rate (>75%).

### Results

We conducted the same analysis as described in Experiment 1. The results are shown in the middle panel of Figure 2. RT data refer to RTs post-stimulus offset. The ANOVA on the accuracy data revealed a main effect of bodily emotion (*F*_(1,18)_ = 10.174; *p* = 0.005; $\eta_{p}^{2}=0.361$) and body × face emotion interaction (*F*_(1,18)_ = 12.455; *p* = 0.002; $\eta_{p}^{2}=0.409$). The main effect of body expression indicates that faces with a neutral body are more accurately matched than faces with a fearful body. To follow up on the interaction, we quantified the effect of body emotion (neutral body minus fearful body) as a function of face emotion. A paired sample *t*-test showed that the body emotion effect was significantly larger for neutral faces (*t*_(18)_ = 3.529, *p* = 0.002). More specifically, fearful bodies result in lower accuracies, but only when they are presented with a neutral face (*t*_(18)_ = 4.328; *p* < 0.001) and not with a fearful face (*t*_(18)_ = 0.475; *p* = 0.640). The analysis of the reaction times revealed a main effect of facial emotion (*F*_(1,18)_ = 13.552, *p* = 0.002; $\eta_{p}^{2}=0.430$) as the only significant result, with fearful faces resulting in longer RTs than neutral faces.

### Discussion

The results of Experiment 2 show that the body expression effect also holds when the viewing time is shortened to 750 ms in order to minimize visual exploration of the task irrelevant body expression. Moreover, a pilot study showed that 750 ms is the minimal duration to obtain an overall accuracy of at least 75% (when chance level is 50%). This result indicates that the body expression effect cannot fully be explained by extensive visual exploration of the fearful body expressions, compared to the neutral body expressions. Although 750 ms was the shortest duration at which participants showed a satisfactory performance according to the results of the pilot study, this duration does not exclude a differential looking time at fearful vs. neutral bodies.

In addition, the results indicate that the body expression effect primarily occurs when the facial expression is neutral, consistent with our previous study on the influence of body expressions on categorization of facial expressions (Van den Stock et al., 2007).

In both Experiments 1 and 2, participants had to make a saccade from the face on top to the two faces at the bottom of the stimulus. The area spanning the distance between the two fixation regions contains the bodily expression, which raises the question whether the effects can be explained by the fact that a saccade always covers the region of the body expression. To investigate this issue, we modified the design in order to exclude saccades across the body expression in Experiment 3.

## Experiment 3: time-constrained delayed matching of face identity

### Method

#### Participants

Nineteen participants volunteered for the experiment (14 male, mean (SD) age = 19.8 (1.9)) in exchange for course credits. None of the participants had a neurologic or psychiatric history and all had normal or corrected to normal vision. Informed consent was obtained according to the declaration of Helsinki.

#### Procedure

The procedure was identical to the one in Experiment 1, except that the task was modified to a delayed match-to-sample task. The face-body compound was presented for 150 ms, which is insufficient to encode the face and make a saccade. A 5000 ms delay during which a blank screen was presented, followed the stimulus. We included this delay, to avoid responses based on after-images. Subsequently, the two isolated faces were presented until the participant responded. This design does not require any saccades of the subject during presentation of the face-body compound stimulus and minimizes the occurrence of after-image effects.

While we could also have moved the answer stimuli above the central display to avoid saccades, we preferred to make a more substantial change to the design, while maintaining the central research question (does body emotion influence processing of face identity?). Furthermore, the 150 ms presentation of the composite stimulus does not provide enough time to look at the task irrelevant body as well as sufficiently encoding the identity of the face stimulus. It should be stated that the task required that the identity was sufficiently encoded and stored in working memory, as the response screen did not appear until 5000 ms after the offset of the composite stimulus.

### Results

The results are shown in the right panel of Figure 2. RT data refer to RTs measured from the onset of the screen showing the two face images. The analysis of the accuracy data revealed a main effect of body expression (*F*_(1,18)_ = 8.824, *p* = 0.008; $\eta_{p}^{2}=0.329$), while there was a main effect of body (*F*_(1,18)_ = 6.958, *p* = 0.017; $\eta_{p}^{2}=0.279$) and face expression (*F*_(1,18)_ = 5.449, *p* = 0.031; $\eta_{p}^{2}=0.232$) in the RT data. The main effects of body expression reflect the fact that faces combined with a neutral body are matched faster and more accurate than faces with a fearful body, while the main effect of facial expression indicates that neutral faces are matched faster than fearful faces.

### Discussion

The results show that sequential matching of face identity is influenced by the task irrelevant body expression, even when presentation time is reduced to 150 ms, no saccades are required and the influence of after-image effects are minimized.

## Between-Experiments analysis

To investigate the effect of the three experimental designs, we performed a repeated-measures ANOVA with version as between-subjects variable (self-paced direct matching; time constrained direct matching; delayed matching) and facial expression and body expression as within-subject variables on the accuracy and the reaction time data. For the accuracy data, the results revealed a significant main effect of body expression (*F*_(1,55)_ = 23.878, *p* < 0.001; $\eta_{p}^{2}=0.303$), reflecting lower performance for fearful body expressions; a significant main effect of version (*F*_(2,55)_ = 8.686, *p* < 0.001; $\eta_{p}^{2}=0.240$), a significant body expression × face expression interaction (*F*_(1,55)_ = 4.186, *p* = 0.046; $\eta_{p}^{2}=0.071$) and finally a significant body expression × face expression × version interaction (*F*_(2,55)_ = 4.560, *p* = 0.015; $\eta_{p}^{2}=0.142$). Bonferroni corrected post-hoc tests on the main and interaction effects revealed that accuracies were higher in Experiment 1 (self-paced) than in Experiment 2 (*p* = 0.004) and Experiment 3 (*p* = 0.001), while there was no difference between Experiments 2 and 3 (*p* = 0.999). Follow-up of the body expression × face expression interaction by means of a paired *t*-tests showed that the effect of the body emotion (neutral body minus fearful body) was larger for neutral faces than for fearful faces, although this was only marginally significant (*t*_(57)_ = 1.877, *p* = 0.066). More specifically, a fearful body expression only significantly reduced performance when the face was neutral (*t*_(57)_ = 4.096, *p* < 0.001) but not when the face was fearful (*t*_(57)_ = 1.327, *p* = 0.379). Similarly, a fearful face expression only reduced performance when the body was neutral (*t*_(57)_ = 2.152, *p* = 0.036) and not when the body was fearful (*t*_(57)_ = 0.596, *p* = 0.553). We performed a one-way ANOVA with Experiment (3 levels) as factor on the differential effect of body emotion on face emotion ((neutral face/neutral body minus neutral face/fearful body) minus (fearful face/neutral body minus fearful face/fearful body)). This revealed a main effect (*F*_(2,57)_ = 4.560, *p* = 0.015) and Tukey Honestly Significant Difference (HSD) corrected *post-hoc* tests showed that there was only a significant difference between Experiment 1 and 2, indicating that the body emotion × face emotion interaction effect was larger in Experiment 2 than in Experiment 1. For the reaction time data, there was a main effect of body expression (*F*_(1,55)_ = 21.455, *p* < 0.001; $\eta_{p}^{2}=0.281$) reflecting slower performance for fearful body expressions; a main effect of face expression (*F*_(1,55)_ = 10.500, *p* = 0.002; $\eta_{p}^{2}=0.160$), reflecting slower performance for fearful face expressions; and a main effect of version (*F*_(2,55)_ = 41.670, *p* < 0.001; $\eta_{p}^{2}=0.602$).

## General discussion

Recently we have documented that recognition memory for face identity is influenced by the affective valence of the visual context, as conveyed by body expressions (Van den Stock and de Gelder, 2012). We hypothesized that these differences originate at the perception stage and therefore predicted for the current study that matching of facial identity is influenced by the emotional context, i.e., body expressions (de Gelder and Bertelson, 2003).

We performed three experiments investigating the influence of task irrelevant body and face expressions on processing of facial identity. Participants were presented realistic face-body compounds in a 2 category (face and body) × 2 emotion (neutral and fearful) factorial design. The task always consisted of two-alternative forced choice facial identity matching. Although the task variables were increasingly manipulated to tap into facial identity processing and aimed to minimize effects of non-interest, such as simple image matching, viewing time and attention, there was always an influence of the task irrelevant body expression. Moreover, the analysis of the pooled data of the three experiments revealed that the most significant and largest effect was the effect of body emotion.

There is evidence showing that both faces and bodies share similar perceptual (Robbins and Coltheart, 2012) and neural (Reed et al., 2003; Stekelenburg and de Gelder, 2004; Van De Riet et al., 2009; Schmalzl et al., 2012) processing routines and this may be the underlying mechanism through which face-body interactions occur. In fact, a similar mechanism has been proposed for facial expression recognition (Van den Stock et al., 2007) and recent data indicate that disrupting the canonical face-body configuration, reduces the influence of the body expression on the recognition of the facial expression (Aviezer et al., 2012). Although accumulating evidence shows that both faces and bodies are processed configurally, this does not exclude that a face-body compound stimulus is processed as one configuration. In fact, an event-related potential (ERP)-study showed that the emotional expression of a body influences the early electrophysiological markers (P1, occurring around 115 ms) during facial expression categorization (Meeren et al., 2005). Perhaps the strongest behavioral support for the hypothesis that processing of the identity of a face has a strong intrinsic coupling with the body is provided in a recent study revealing that adaptation to body identity results in perceptual after-effects on facial identity perception (Ghuman et al., 2010).

Alternatively, it cannot be ruled out that a fearful body expression attracted more (covert) attention (Posner and Petersen, 1990) than the neutral body posture (Bannerman et al., 2009). In line with this there is evidence from cortically blind patients indicating that body shape and body emotion is processed even without awareness (Tamietto et al., 2009; Van den Stock et al., 2011, 2013a). Orienting responses may be triggered by the emotional body expression in order to detect the source of potential danger, leading to a reduced encoding of facial details (Kensinger et al., 2007). This could lead to a reduction in time used to process facial identity when combined with a fearful body expression, which could account for the results we report here.

These hypotheses both have adaptive benefits at face value. In the face of danger (as communicated by fearful conspecifics), the primary focus would be to detect and adequately react to the source of danger, rather than devoting resources to the processing of the identity of the bystanders. In fact, we have previously provided evidence for a neural mechanism supporting motor preparation when viewing fearful body expressions (de Gelder et al., 2004). The finding that the body expression effect is primarily observed with neutral faces is compatible with this line of reasoning. When the stimulus at the focus of attention, i.e., the face, is signaling threat, the body expression is of less importance and has less influence. By extension, the present results provide evidence that the interactions between face identity and face emotion processing that have been previously reported (D’argembeau et al., 2003; Kaufmann and Schweinberger, 2004; Gallegos and Tranel, 2005; D’argembeau and Van Der Linden, 2007; Savaskan et al., 2007; Levy and Bentin, 2008; Chen et al., 2011) also apply for face identity and body expression.

However, in the analysis of the accuracy data of the combined Experiments, there was a main effect of body expression, while the effect of face expression only occurred in interaction with the body expression. The interaction effect more particularly revealed that the effect of body expression was significantly larger when the face expression was neutral and similarly, the effect of face expression only occurred when the body expression was neutral. The absence of a main effect of face expression, in combination with the occurrence of the main effect of body expression and face × body expression interaction may reflect that the body expression influence outweighs the influence of facial emotion on face identity matching. This conjecture would be in line with fMRI-studies, directly comparing emotional face and body stimuli. While faces typically trigger more amygdala and striate cortex activity compared to bodies, the inverse contrast appears to activate a more widespread and extensive set of regions, including frontal, parietal, temporal, occipital and subcortical structures (Van De Riet et al., 2009; Kret et al., 2011).

Although cross-categorical influences on emotion recognition have been mainly examined at the perception stage, the neural correlates of emotional influence on identity recognition have been primarily investigated in the memory stage and the findings point to an important role of the amygdala (for reviews, see Hamann, 2001; Kensinger, 2004; Phelps, 2004; Labar and Cabeza, 2006). The amygdala may also play a role in the effects we observe in the present study. It has been documented that both neutral and fearful faces activate the amygdala (Zald, 2003; Fusar-Poli et al., 2009), as well as fearful and neutral body expressions (Hadjikhani and de Gelder, 2003; de Gelder et al., 2004, 2010; Van den Stock et al., 2014). In addition, we have shown that emotional body expressions presented in the blind hemifield of a cortically blind patient activates the amygdala as well as other subcortical structures like colliculus superior and the thalamic pulvinar (de Gelder and Hadjikhani, 2006; Van den Stock et al., 2011). These findings support the notion that emotional body expressions are processed automatically and thereby have an influence on face identity perception.

The current study supports the notion that the effects of body expression on recognition memory for face identity (Van den Stock and de Gelder, 2012) originate at least in part during the perception stage. In Experiment 1 we used a rather “liberal” set-up with unlimited viewing time and participants were instructed to respond as accurate and quickly as possible. Although the average reaction time was around 1500 ms, the accuracy data showed no ceiling effect. This finding may be explained by the fact that participants engaged in visual exploration of the task irrelevant body expression.

In Experiment 2, stimulus presentation of the face-body compound was limited to 750 ms, which was the minimal duration to allow sufficient accuracy (>75%) on the basis of a pilot study. Although we have no objective measure that participants refrained from looking at the body expression, the short presentation of the compound stimulus does not allow elaborate exploration of the body expression. The average reaction time of about 1200 ms (750 ms stimulus presentation + around 450 ms response latency) is about 300 ms shorter than in Experiment 1 and compatible with the notion that participants spent more time looking at the body expression in Experiment 1.

However, in both Experiments 1 and 2, the task required making a saccade across the body expression. This was no longer the case in Experiment 3, which also reduced presentation of the compound stimulus to 150 ms, which is insufficient to visually explore the task irrelevant body expression. Interestingly, the results still showed an influence of the body expression on face identity processing.

In conclusion, the results of the present study indicate that task irrelevant bodily expressions influence facial identity matching under different task conditions and hence the findings are compatible with an automatic interaction of emotional expression information and face identity processing.

## Conflict of interest statement

The authors declare that the research was conducted in the absence of any commercial or financial relationships that could be construed as a potential conflict of interest.
